# Crystal structure of (*E*)-*N*-phenyl-*N*′-[1-(thio­phen-2-yl)ethyl­idene]formo­hydrazide

**DOI:** 10.1107/S1600536814016511

**Published:** 2014-08-01

**Authors:** C. S. Dileep, K. R. Raghavendra, N. K. Lokanath, K. Ajay Kumar, M. A. Sridhar

**Affiliations:** a Department of Studies in Physics, Manasagangotri, University of Mysore, Mysore 570 006, India; b Department of Chemistry, SBRR Mahajana College, Mysore 570 006, India; c Post Graduate Department of Chemistry, Yuvaraja College, University of Mysore, Mysore 570 005, India

**Keywords:** crystal structure, thio­phene derivative, hydrogen bonding

## Abstract

In the title compound, C_13_H_12_N_2_OS, the planes of the thio­phene and phenyl rings are nearly perpendicular to each other, making a dihedral angle of 86.42 (12)°. In the crystal, mol­ecules are linked by C—H⋯O hydrogen bonds, forming a helical chain along the *b*-axis direction.

## Related literature   

For the biological activity of thio­phene derivatives, see: Bondock *et al.* (2010 ▶); Bellina *et al.* (2007 ▶); Konstanti­nova *et al.* (2009 ▶); Al-Said *et al.* (2011 ▶). For the crystal structure of a similar compound, *viz.* (*E*)-*N*′-[1-(thio­phen-2-yl)ethyl­idene]benzohydrazide, see: Shan *et al.* (2011 ▶). For a description of the Cambridge Structural Database, see: Allen (2002 ▶).
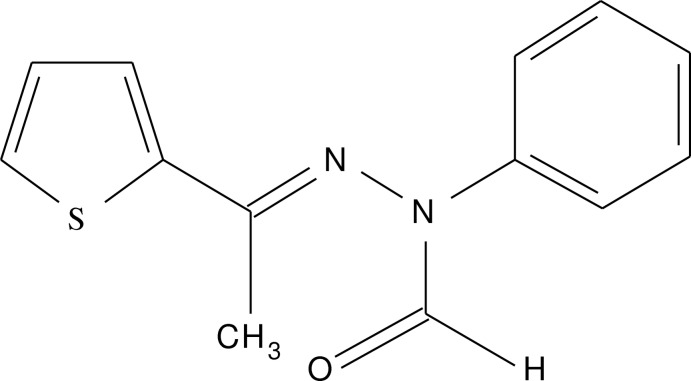


## Experimental   

### Crystal data  

C_13_H_12_N_2_OS
*M* *_r_* = 244.32
Orthorhombic, 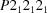

*a* = 5.4960 (7) Å
*b* = 11.0177 (13) Å
*c* = 20.249 (2) Å
*V* = 1226.1 (2) Å^3^
*Z* = 4
Cu *K*α radiation
μ = 2.22 mm^−1^
*T* = 296 K
0.25 × 0.22 × 0.20 mm

### Data collection  

Bruker X8 Proteum diffractometer
Absorption correction: multi-scan (*SADABS*; Bruker, 2013 ▶) *T*_min_ = 0.604, *T*_max_ = 0.662
6298 measured reflections
2010 independent reflections
1904 reflections with *I* > 2σ(*I*)
*R*_int_ = 0.042

### Refinement  

*R*[*F* ^2^ > 2σ(*F* ^2^)] = 0.038
*wR*(*F* ^2^) = 0.102
*S* = 1.10
2010 reflections
165 parameters
H-atom parameters constrained
Δρ_max_ = 0.21 e Å^−3^
Δρ_min_ = −0.20 e Å^−3^
Absolute structure: Flack (1983 ▶), 805 Friedel pairs
Absolute structure parameter: 0.02 (2)

### 

Data collection: *APEX2* (Bruker, 2013 ▶); cell refinement: *SAINT* (Bruker, 2013 ▶); data reduction: *SAINT*; program(s) used to solve structure: *SHELXS97* (Sheldrick, 2008 ▶); program(s) used to refine structure: *SHELXL97* (Sheldrick, 2008 ▶); molecular graphics: *Mercury* (Macrae *et al.*, 2006 ▶); software used to prepare material for publication: *PLATON* (Spek, 2009 ▶).

## Supplementary Material

Crystal structure: contains datablock(s) global, I. DOI: 10.1107/S1600536814016511/is5369sup1.cif

Structure factors: contains datablock(s) I. DOI: 10.1107/S1600536814016511/is5369Isup2.hkl

Supporting information file. DOI: 10.1107/S1600536814016511/is5369Isup3.cml

**Fig. 1.**
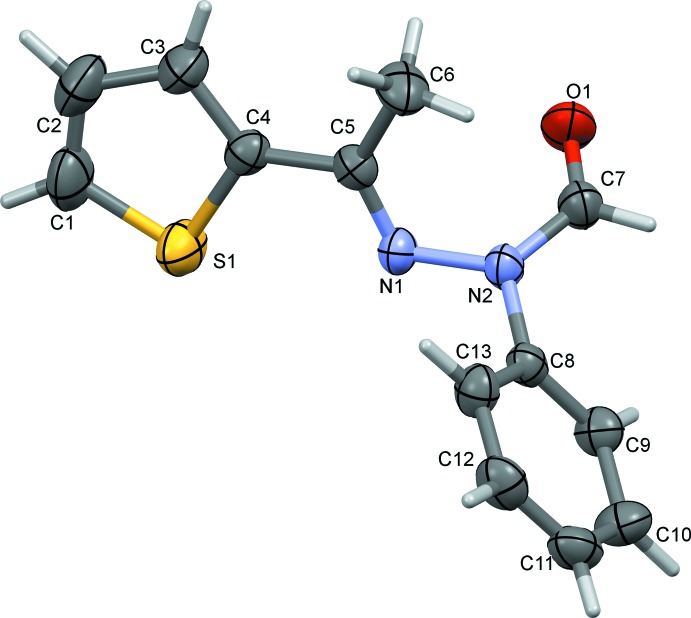
ORTEP view of the molecule with the atom-labeling scheme. The displacement ellipsoids are drawn at the 50% probability level.

**Fig. 2.**
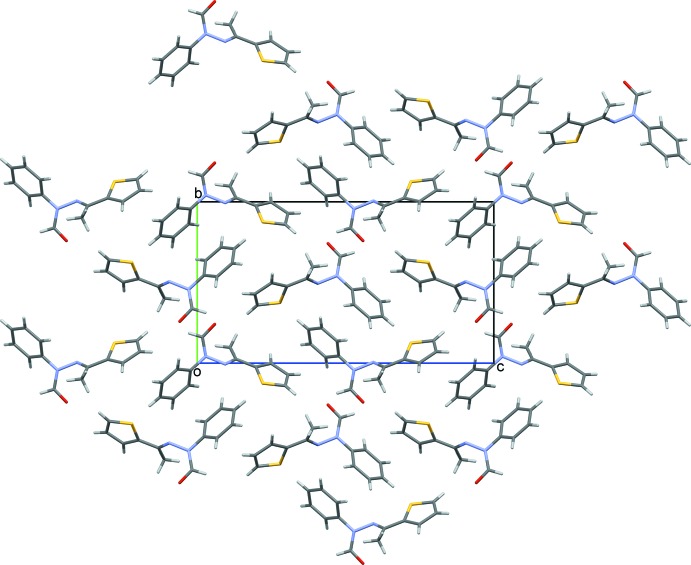
A molecular packing view of the title compound down the a-axis.

ORTEP . DOI: 10.1107/S1600536814016511/is5369fig1.tif
*ORTEP* view of the mol­ecule with the atom-labeling scheme. The displacement ellipsoids are drawn at the 50% probability level.

a . DOI: 10.1107/S1600536814016511/is5369fig2.tif
A mol­ecular packing view of the title compound down the *a*-axis.

CCDC reference: 1014287

Additional supporting information:  crystallographic information; 3D view; checkCIF report

## Figures and Tables

**Table 1:** Hydrogen-bond geometry (Å, °)

*D*—H⋯*A*	*D*—H	H⋯*A*	*D*⋯*A*	*D*—H⋯*A*
C1—H1⋯O1^i^	0.93	2.39	3.202 (3)	145

Symmetry code: (i) 
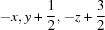
.

## supplementary crystallographic information

### S1. Comment

In medicinal chemistry, thiophene derivatives have been very well known for their therapeutic applications. Many thiophene derivatives have been developed as chemotherapeutic agents and are extensively used. Thiophene nucleus is one of the most important heterocycles exhibiting remarkable pharmacological activities. The great interest in the synthesis of thiophene derivatives due to their diverse biological and chemical properties. Thiophene, as a prominent structural motif, is found in numerous active compounds, which contain 5-membered heterocyclic structure have attracted a lot of interests in many fields, and its rich biological activity in medicinal chemistry owing to their biological properties. Thiophene and thiazole derivatives are known to possess interesting biological properties like anticancer (Bondock *et al.*, 2010; Bellina *et al.*, 2007; Konstantinova *et al.*, 2009). Thiophene or benzothiophene moieties due to the well documented anti-cancer activity of these moieties to study their SAR and their anti-breast cancer activity (Al-Said *et al.*, 2011). In view of their importance as discussed above, thiophene derivatives were taken for their conformational studies to get better structural activity correlation.

In the title compound (Fig. 1), the bond lengths do not show much variation in the core structure of the derivatives, and are similar to the standard values (Allen *et al.*, 2002). The thiophene (S1/C1–C4) and phenyl (C8–C13) rings are nearly perpendicular with a dihedral angle of 86.42 (12)° between their mean planes. The bond lengths and bond angles do not show large deviations and are comparable with those reported for a similar structure (Shan *et al.*, 2011). The conformation of the attachment of the thiophene and phenyl rings can also be characterized by torsion angles of (C4—C5—N1—N2), (C5—N1—N2—C8), (O1—C7—N2—C8) and (S1—C4—C5—C6) being 178.38, 127.73, 171.34 and -170.31°, respectively. The crystal structure has an intermolecular C—H···O hydrogen bond. The molecular packing viewed down the *a* axis is shown in Fig. 2.

### S2. Experimental

A mixture of (*E*)-1-phenyl-2-[(1-thiophen-2-yl)ethylidene]hydrazine (0.176 mmol) were added to the Vilsmeier-Haack reagent prepared by drop-wise addition of POCl_3_ (1.2 ml) in ice cooled DMF (5 ml). The mixture was stirred at 60–65 °C for 6 h. The progress of the reaction was monitored by TLC. After completion of the reaction, the mixture was poured into ice cold water, neutralized with NaHCO_3_, the solid separated was filtered, washed with water and recrystallized from ethanol to get the compound in 93% yield.

### S3. Refinement

All H atoms were located from difference maps and were positioned geometrically and refined using a riding model with C—H = 0.93–0.96 Å and *U*_iso_(H) = 1.2*U*_eq_(C).

### Crystal data

**Table:**

C_13_H_12_N_2_OS	*F*(000) = 512
*M**_r_* = 244.32	*D*_x_ = 1.324 Mg m^−^^3^
Orthorhombic, *P*2_1_2_1_2_1_	Cu *K*α radiation, λ = 1.54178 Å
Hall symbol: P 2ac 2ab	Cell parameters from 6298 reflections
*a* = 5.4960 (7) Å	θ = 4.4–64.6°
*b* = 11.0177 (13) Å	µ = 2.22 mm^−^^1^
*c* = 20.249 (2) Å	*T* = 296 K
*V* = 1226.1 (2) Å^3^	Block, pale yellow
*Z* = 4	0.25 × 0.22 × 0.20 mm

### Data collection

**Table:**

Bruker X8 Proteum diffractometer	2010 independent reflections
Radiation source: Bruker MicroStar microfocus rotating anode	1904 reflections with *I* > 2σ(*I*)
Helios multilayer optics monochromator	*R*_int_ = 0.042
Detector resolution: 10.7 pixels mm^-1^	θ_max_ = 64.6°, θ_min_ = 4.4°
φ and ω scans	*h* = −2→6
Absorption correction: multi-scan (*SADABS*; Bruker, 2013)	*k* = −12→12
*T*_min_ = 0.604, *T*_max_ = 0.662	*l* = −23→22
6298 measured reflections	

### Refinement

**Table:**

Refinement on *F*^2^	Hydrogen site location: inferred from neighbouring sites
Least-squares matrix: full	H-atom parameters constrained
*R*[*F*^2^ > 2σ(*F*^2^)] = 0.038	*w* = 1/[σ^2^(*F*_o_^2^) + (0.0605*P*)^2^ + 0.0861*P*] where *P* = (*F*_o_^2^ + 2*F*_c_^2^)/3
*wR*(*F*^2^) = 0.102	(Δ/σ)_max_ = 0.001
*S* = 1.10	Δρ_max_ = 0.21 e Å^−^^3^
2010 reflections	Δρ_min_ = −0.20 e Å^−^^3^
165 parameters	Extinction correction: *SHELXL*, FC^*^=KFC[1+0.001XFC^2^Λ^3^/SIN(2Θ)]^-1/4^
0 restraints	Extinction coefficient: 0.0158 (16)
Primary atom site location: structure-invariant direct methods	Absolute structure: Flack (1983), 805 Friedel pairs
Secondary atom site location: difference Fourier map	Absolute structure parameter: 0.02 (2)

### Special details

**Table:**

Geometry. Bond distances, angles *etc*. have been calculated using the rounded fractional coordinates. All su's are estimated from the variances of the (full) variance-covariance matrix. The cell e.s.d.'s are taken into account in the estimation of distances, angles and torsion angles
Refinement. Refinement on *F*^2^ for ALL reflections except those flagged by the user for potential systematic errors. Weighted *R*-factors *wR* and all goodnesses of fit *S* are based on *F*^2^, conventional *R*-factors *R* are based on *F*, with *F* set to zero for negative *F*^2^. The observed criterion of *F*^2^ > σ(*F*^2^) is used only for calculating -*R*-factor-obs *etc*. and is not relevant to the choice of reflections for refinement. *R*-factors based on *F*^2^ are statistically about twice as large as those based on *F*, and *R*-factors based on ALL data will be even larger.

### Fractional atomic coordinates and isotropic or equivalent isotropic displacement parameters (Å^2^)

**Table:**

	*x*	*y*	*z*	*U*_iso_*/*U*_eq_	
S1	0.05502 (12)	0.12455 (6)	0.72104 (3)	0.0539 (2)	
O1	0.0434 (4)	−0.24121 (15)	0.55887 (8)	0.0526 (6)	
N1	0.0078 (3)	0.00557 (16)	0.59394 (8)	0.0348 (5)	
N2	0.0086 (3)	−0.04249 (15)	0.52882 (8)	0.0344 (5)	
C1	−0.0682 (6)	0.1399 (3)	0.79702 (12)	0.0623 (10)	
C2	−0.2635 (6)	0.0712 (3)	0.80591 (13)	0.0648 (10)	
C3	−0.3227 (5)	0.0016 (2)	0.74907 (11)	0.0513 (8)	
C4	−0.1639 (4)	0.02325 (18)	0.69777 (10)	0.0358 (6)	
C5	−0.1724 (4)	−0.02503 (18)	0.63061 (10)	0.0331 (6)	
C6	−0.3905 (4)	−0.0972 (2)	0.61062 (13)	0.0509 (8)	
C7	0.0373 (4)	−0.16179 (19)	0.51733 (10)	0.0410 (7)	
C8	0.0373 (4)	0.04484 (17)	0.47710 (9)	0.0325 (6)	
C9	0.2273 (4)	0.0366 (2)	0.43330 (11)	0.0397 (6)	
C10	0.2429 (4)	0.1192 (2)	0.38203 (12)	0.0479 (7)	
C11	0.0710 (5)	0.2091 (2)	0.37516 (11)	0.0469 (7)	
C12	−0.1151 (5)	0.2185 (2)	0.41975 (12)	0.0463 (8)	
C13	−0.1322 (4)	0.1365 (2)	0.47156 (11)	0.0413 (6)	
H1	−0.00560	0.19130	0.82930	0.0750*	
H2	−0.35200	0.06880	0.84500	0.0780*	
H3	−0.45300	−0.05220	0.74680	0.0620*	
H6A	−0.39060	−0.10770	0.56360	0.0760*	
H6B	−0.53550	−0.05510	0.62380	0.0760*	
H6C	−0.38540	−0.17530	0.63170	0.0760*	
H7	0.05420	−0.18570	0.47350	0.0490*	
H9	0.34410	−0.02390	0.43800	0.0480*	
H10	0.37060	0.11400	0.35200	0.0570*	
H11	0.08130	0.26340	0.34010	0.0560*	
H12	−0.23010	0.27990	0.41530	0.0560*	
H13	−0.25720	0.14340	0.50230	0.0500*	

### Atomic displacement parameters (Å^2^)

**Table:**

	*U*^11^	*U*^22^	*U*^33^	*U*^12^	*U*^13^	*U*^23^
S1	0.0546 (4)	0.0703 (5)	0.0368 (3)	−0.0147 (3)	−0.0029 (3)	−0.0071 (3)
O1	0.0729 (11)	0.0375 (9)	0.0474 (9)	0.0049 (8)	0.0049 (8)	0.0096 (7)
N1	0.0402 (10)	0.0379 (9)	0.0263 (8)	−0.0024 (8)	−0.0012 (7)	−0.0037 (6)
N2	0.0452 (10)	0.0313 (8)	0.0266 (8)	0.0006 (8)	−0.0005 (7)	−0.0022 (7)
C1	0.085 (2)	0.0689 (17)	0.0330 (12)	0.0037 (16)	−0.0059 (13)	−0.0095 (11)
C2	0.0757 (18)	0.0819 (19)	0.0368 (13)	0.0055 (16)	0.0152 (13)	0.0000 (13)
C3	0.0524 (14)	0.0590 (15)	0.0426 (13)	−0.0041 (12)	0.0127 (11)	−0.0022 (11)
C4	0.0373 (10)	0.0370 (11)	0.0332 (10)	0.0038 (9)	0.0009 (8)	0.0026 (8)
C5	0.0317 (10)	0.0314 (10)	0.0362 (11)	0.0037 (8)	−0.0030 (8)	0.0008 (8)
C6	0.0372 (12)	0.0564 (15)	0.0592 (14)	−0.0111 (10)	0.0008 (11)	−0.0119 (12)
C7	0.0511 (12)	0.0353 (11)	0.0366 (11)	0.0020 (9)	0.0024 (10)	−0.0018 (9)
C8	0.0388 (10)	0.0296 (10)	0.0291 (9)	−0.0017 (8)	−0.0051 (8)	−0.0012 (8)
C9	0.0355 (10)	0.0392 (11)	0.0444 (12)	0.0049 (9)	0.0011 (9)	0.0016 (10)
C10	0.0456 (12)	0.0511 (13)	0.0469 (13)	−0.0056 (11)	0.0089 (10)	0.0094 (11)
C11	0.0604 (14)	0.0365 (11)	0.0439 (12)	−0.0080 (11)	−0.0054 (11)	0.0088 (9)
C12	0.0536 (14)	0.0350 (12)	0.0503 (13)	0.0084 (11)	−0.0087 (11)	0.0024 (10)
C13	0.0429 (11)	0.0416 (11)	0.0395 (11)	0.0083 (10)	0.0012 (10)	−0.0035 (10)

### Geometric parameters (Å, º)

**Table:**

S1—C1	1.690 (3)	C10—C11	1.376 (3)
S1—C4	1.707 (2)	C11—C12	1.368 (4)
O1—C7	1.214 (3)	C12—C13	1.388 (3)
N1—N2	1.421 (2)	C1—H1	0.9300
N1—C5	1.283 (3)	C2—H2	0.9300
N2—C7	1.344 (3)	C3—H3	0.9300
N2—C8	1.431 (2)	C6—H6A	0.9600
C1—C2	1.326 (5)	C6—H6B	0.9600
C2—C3	1.421 (4)	C6—H6C	0.9600
C3—C4	1.378 (3)	C7—H7	0.9300
C4—C5	1.461 (3)	C9—H9	0.9300
C5—C6	1.494 (3)	C10—H10	0.9300
C8—C9	1.373 (3)	C11—H11	0.9300
C8—C13	1.379 (3)	C12—H12	0.9300
C9—C10	1.383 (3)	C13—H13	0.9300
			
C1—S1—C4	91.96 (13)	C2—C1—H1	123.00
N2—N1—C5	116.20 (17)	C1—C2—H2	124.00
N1—N2—C7	121.70 (16)	C3—C2—H2	124.00
N1—N2—C8	115.40 (15)	C2—C3—H3	124.00
C7—N2—C8	121.19 (16)	C4—C3—H3	124.00
S1—C1—C2	113.0 (2)	C5—C6—H6A	109.00
C1—C2—C3	112.6 (2)	C5—C6—H6B	109.00
C2—C3—C4	111.9 (2)	C5—C6—H6C	109.00
S1—C4—C3	110.58 (16)	H6A—C6—H6B	109.00
S1—C4—C5	121.16 (16)	H6A—C6—H6C	109.00
C3—C4—C5	128.2 (2)	H6B—C6—H6C	109.00
N1—C5—C4	114.73 (19)	O1—C7—H7	117.00
N1—C5—C6	127.04 (19)	N2—C7—H7	117.00
C4—C5—C6	118.13 (19)	C8—C9—H9	120.00
O1—C7—N2	126.02 (19)	C10—C9—H9	120.00
N2—C8—C9	120.81 (18)	C9—C10—H10	120.00
N2—C8—C13	118.53 (18)	C11—C10—H10	120.00
C9—C8—C13	120.65 (19)	C10—C11—H11	120.00
C8—C9—C10	119.2 (2)	C12—C11—H11	120.00
C9—C10—C11	120.5 (2)	C11—C12—H12	120.00
C10—C11—C12	120.1 (2)	C13—C12—H12	120.00
C11—C12—C13	120.0 (2)	C8—C13—H13	120.00
C8—C13—C12	119.5 (2)	C12—C13—H13	120.00
S1—C1—H1	124.00		
			
C4—S1—C1—C2	0.9 (3)	C2—C3—C4—C5	−176.1 (2)
C1—S1—C4—C3	−1.3 (2)	C2—C3—C4—S1	1.4 (3)
C1—S1—C4—C5	176.4 (2)	S1—C4—C5—N1	6.3 (3)
C5—N1—N2—C7	−67.0 (2)	C3—C4—C5—N1	−176.4 (2)
C5—N1—N2—C8	127.7 (2)	C3—C4—C5—C6	7.0 (3)
N2—N1—C5—C4	178.38 (16)	S1—C4—C5—C6	−170.31 (16)
N2—N1—C5—C6	−5.3 (3)	N2—C8—C9—C10	176.94 (19)
C7—N2—C8—C13	135.3 (2)	C13—C8—C9—C10	−2.1 (3)
N1—N2—C7—O1	6.9 (3)	N2—C8—C13—C12	−176.7 (2)
C8—N2—C7—O1	171.3 (2)	C9—C8—C13—C12	2.3 (3)
N1—N2—C8—C9	121.6 (2)	C8—C9—C10—C11	0.4 (3)
C7—N2—C8—C9	−43.7 (3)	C9—C10—C11—C12	1.0 (4)
N1—N2—C8—C13	−59.4 (2)	C10—C11—C12—C13	−0.7 (4)
S1—C1—C2—C3	−0.3 (4)	C11—C12—C13—C8	−0.9 (3)
C1—C2—C3—C4	−0.7 (4)		

### Hydrogen-bond geometry (Å, º)

**Table:**

*D*—H···*A*	*D*—H	H···*A*	*D*···*A*	*D*—H···*A*
C1—H1···O1^i^	0.93	2.39	3.202 (3)	145

Symmetry code: (i) −*x*, *y*+1/2, −*z*+3/2.

## References

[bb1] Allen, F. H. (2002). *Acta Cryst.* B**58**, 380–388. 10.1107/s0108768102003890 12037359

[bb2] Al-Said, M. S., Bashandy, M. S., Al-qasoumi, S. I. & Ghorab, M. M. (2011). *Eur. J. Med. Chem.* **46**, 137–141. 10.1016/j.ejmech.2010.10.024 21093116

[bb3] Bellina, F., Cauteruccio, S. & Rossi, R. (2007). *Tetrahedron*, **63**, 4571–4624.

[bb4] Bondock, S., Fadaly, W. & Metwally, M. A. (2010). *Eur. J. Med. Chem.* **45**, 3692–3701. 10.1016/j.ejmech.2010.05.018 20605657

[bb5] Bruker (2013). *APEX2*, *SADABS* and *SAINT* Bruker AXS Inc., Madison, Wisconsin, USA.

[bb6] Flack, H. D. (1983). *Acta Cryst.* A**39**, 876–881.

[bb7] Konstantinova, L. S., Bolshakov, O. I., Obruchnikova, N. V., Laborie, H., Tanga, A., Sopena, V., Lanneluc, I., Picot, L., Sable, S., Thiery, V. & Rakitin, O. A. (2009). *Bioorg. Med. Chem. Lett.* **19**, 136–141. 10.1016/j.bmcl.2008.11.010 19036587

[bb8] Macrae, C. F., Edgington, P. R., McCabe, P., Pidcock, E., Shields, G. P., Taylor, R., Towler, M. & van de Streek, J. (2006). *J. Appl. Cryst.* **39**, 453–457.

[bb9] Shan, S., Huang, Y.-L., Guo, H.-Q., Li, D.-F. & Sun, J. (2011). *Acta Cryst.* E**67**, o2498. 10.1107/S1600536811033101 PMC3200815 22059048

[bb10] Sheldrick, G. M. (2008). *Acta Cryst.* A**64**, 112–122. 10.1107/S0108767307043930 18156677

[bb11] Spek, A. L. (2009). *Acta Cryst.* D**65**, 148–155. 10.1107/S090744490804362X PMC2631630 19171970

