# Supplementary figures and images for: Crystal structure of (E)-N-phenyl-N′-[1-(thio­phen-2-yl)ethyl­idene]formo­hydrazide
Source: Acta Crystallogr Sect E Struct Rep Online. 2014 Aug 1;70(Pt 9):o928–9. doi: 10.1107/S1600536814016511 (PMC4186119; doi:10.1107/S1600536814016511)

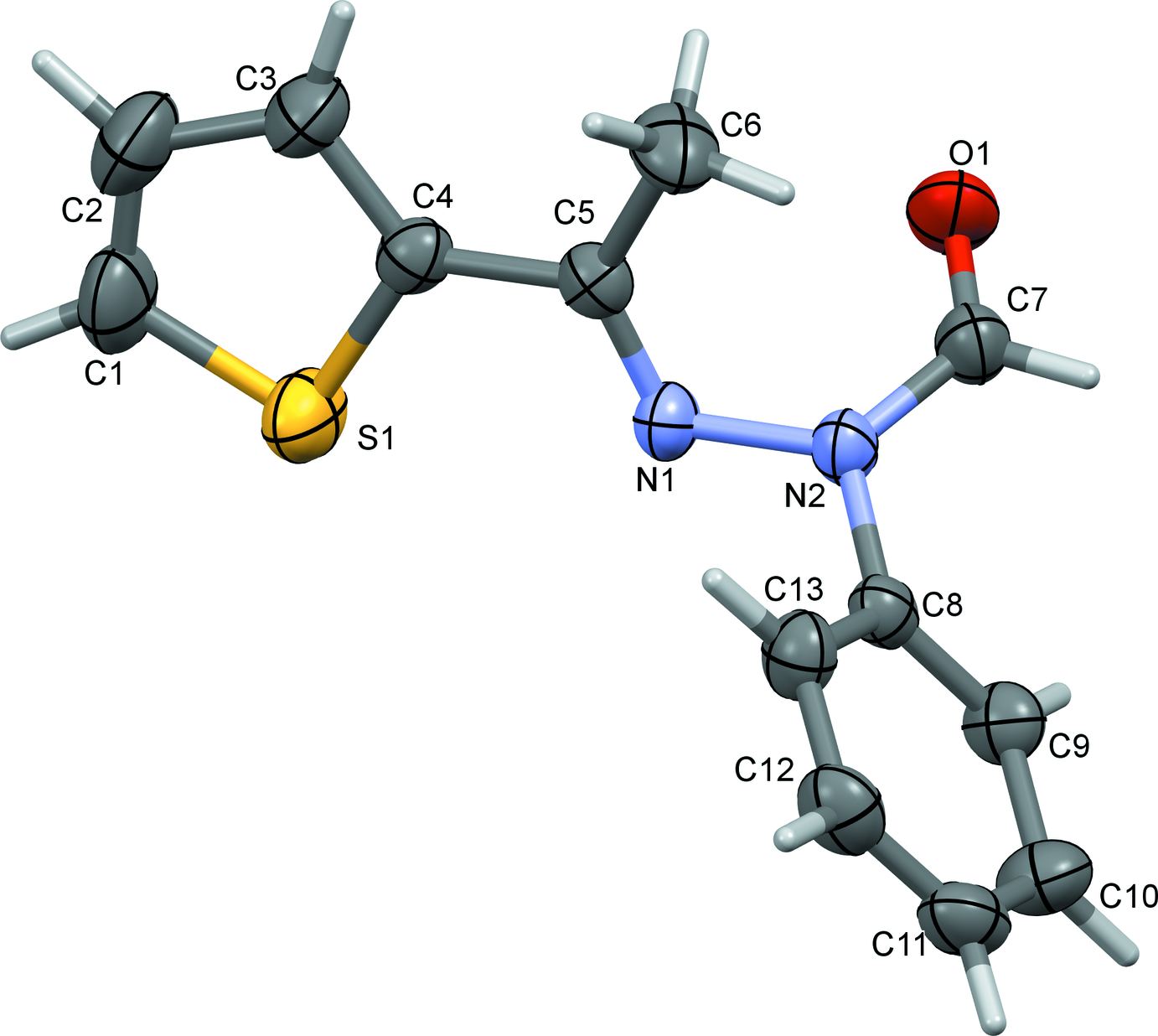

Supplement: Supplementary file 4 [file e-70-0o928-fig1.tif]

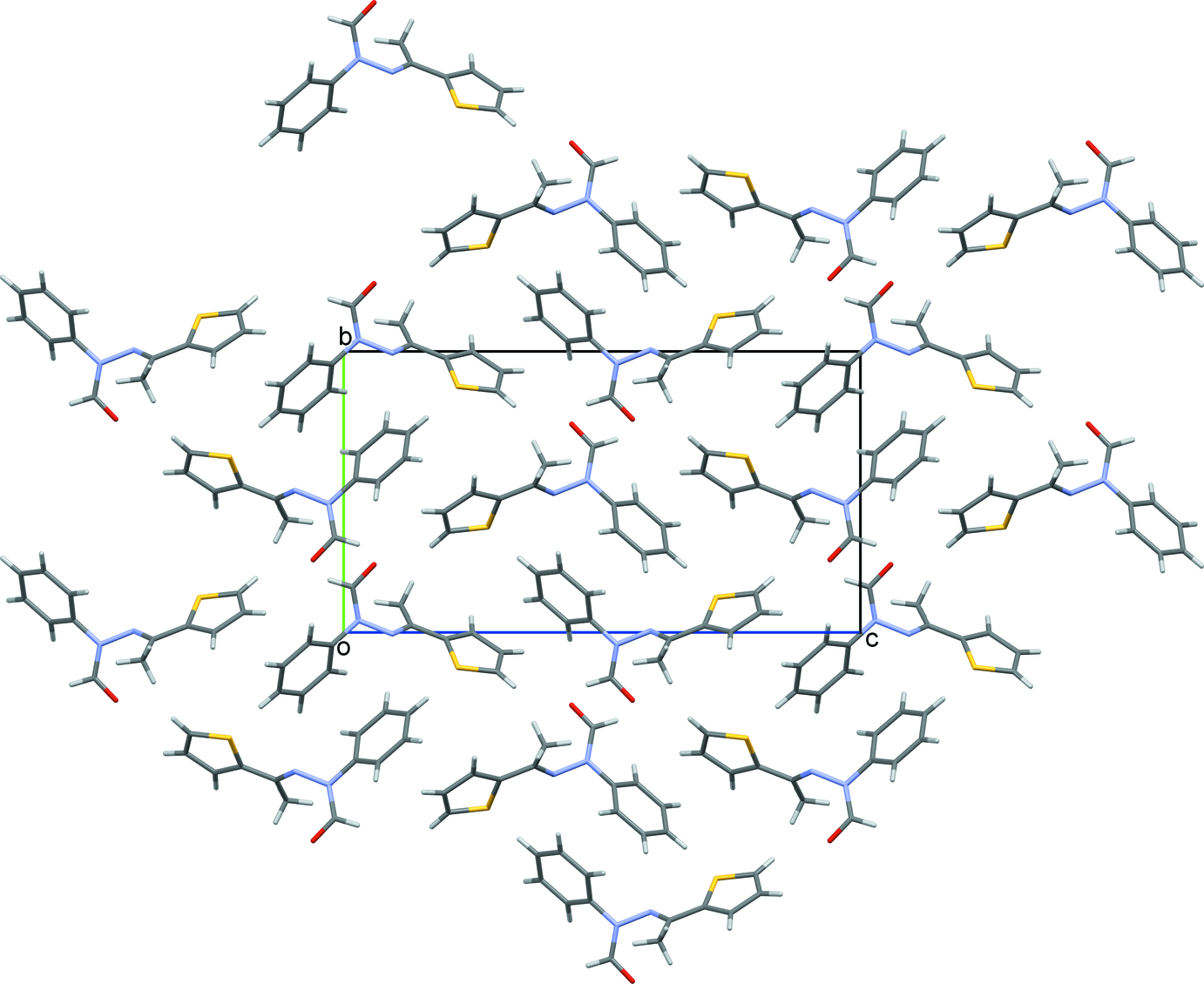

Supplement: Supplementary file 5 [file e-70-0o928-fig2.tif]
